# nNav1.5 expression is associated with glutamate level in breast cancer cells

**DOI:** 10.1186/s40659-022-00387-1

**Published:** 2022-04-29

**Authors:** Irfan Irsyad Azahar, Nur Aishah Sharudin, Ahmad Hafiz Murtadha Noor Din, Ahmad Tarmizi Che Has, Siti Norasikin Mohd Nafi, Hasnan Jaafar, Noor Fatmawati Mokhtar

**Affiliations:** 1 Institute for Research in Molecular Medicine (INFORMM), Universiti Sains Malaysia, Health Campus, 16150 Kubang Kerian, Kelantan Malaysia; 2 Department of Neuroscience, School of Medical Sciences, Universiti Sains Malaysia, Health Campus, 16150 Kubang Kerian, Kelantan Malaysia; 3 Department of Pathology, School of Medical Sciences, Universiti Sains Malaysia, Health, Kelantan Malaysia

**Keywords:** Endogenous glutamate, Exogenous glutamate, Breast cancer, Voltage-gated sodium channels, Neonatal Nav1.5, Invasion

## Abstract

**Background:**

Glutamate and voltage-gated sodium channels, both have been the target of intense investigation for its involvement in carcinogenesis and progression of malignant disease. Breast cancer with increased level of glutamate often metastasize to other organs (especially bone), whilst re-expression of ‘neonatal’ Nav1.5, nNav1.5 in breast cancer is known to promote cell invasion in vitro, metastasis in vivo and positive lymph node metastasis in patients.

**Methods:**

In this study, the role of nNav1.5 in regulating glutamate level in human breast cancer cells was examined using pharmacological approach (VGSCs specific blocker, TTX, glutamate release inhibitor, riluzole and siRNA-nNav1.5). Effect of these agents were evaluated based on endogenous and exogenous glutamate concentration using glutamate fluorometric assay, mRNA expression of nNav1.5 using qPCR and finally, invasion using 3D culture assay.

**Results:**

Endogenous and exogenous glutamate levels were significantly higher in aggressive human breast cancer cells, MDA-MB-231 cells compared to less aggressive human breast cancer cells, MCF-7 and non-cancerous human breast epithelial cells, MCF-10A. Treatment with TTX to MDA-MB-231 cells resulted in significant reduction of endogenous and exogenous glutamate levels corresponded with significant suppression of cell invasion. Subsequently, downregulation of nNav1.5 gene was observed in TTX-treated cells.

**Conclusions:**

An interesting link between nNav1.5 expression and glutamate level in aggressive breast cancer cells was detected and requires further investigation.

## Introduction

Voltage-gated sodium channels (VGSC) are transmembrane protein expressed abundantly in excitable cells such as neurons and muscle cells. In neurons, its main role is to propagate action potential critical in glutamatergic neurotransmission i.e. release of signalling molecules, glutamate at pre-synapses [22]. Interestingly, VGSC and glutamate also characterizes neoplastic cells [10, 13].

Abnormal expression of VGSC in carcinomas (cancer of the epithelial origin) such as cancer of the breast, prostate, lung, cervical, colon and ovarian had been a conundrum to physiologist but subsequent ‘proof-of-concept’ of their critical role in potentiating metastatic cascades using tetrodotoxin (TTX) and other modulating agents, followed by their detectable expression in patient tumour tissues raised their status as a potent metastatic marker [9, 14, 15, 18, 20, 29]. While there are several subtypes of VGSC found upregulated in a number of carcinomas, breast cancer in particular, there is distinctively high expression of the ‘neonatal’ splice variant of the cardiac VGSC isoform, Nav1.5 (nNav1.5) that potentiates motility, migration, and invasion of aggressive human breast cancer cells in vitro and metastasis in vivo [10]. Detectable expression of nNav1.5 in breast tumour tissues positive for lymph node metastasis signified its clinical importance in the prognosis of breast cancer patients [14, 44].

In recent years, several mechanical insights for the role of Nav1.5 in controlling breast cancer cells capacity to metastasize emerged, mainly involve strong influx and elevated concentration of Na^+^ which interferes with Ca^2+^, pH, and gene expression [1]. This mode of interferences has been reported to be associated with the activation of proteases/peptidases activity which enhance degradation of surrounding environment/cells to make way for invasion clearly demonstrated in breast cancer [5, 16, 17]. Accordingly, the opening of Nav1.5 allows strong influx of Na^+^ resulted in elevation of intracellular Na^+^ concentration. A more positive intracellular environment leads to activation of Na^+^ /H^+^ -1 exchanger (NHE1) which allows efflux of H^+^ resulted in extracellular acidification from accumulation of proton. Lower pH at the extracellular microenvironment activates cysteine cathepsins B and S that degrades extracellular matrix, favouring cell invasion [5, 16, 17]. Unfortunately, this type of mechanical insight data for nNav1.5 in breast cancer is poorly understood.

On the same note, elevated glutamate levels in the extracellular environment of rapidly-growing glioblastoma actively kill the surrounding cells to create space for invasion [37]. Glutamate secretion have also been observed in cancer cell lines and tumour tissues of non-neuronal/central nervous system origin [19, 21, 34, 38–40]. Accordingly, elevated glutamate levels in prostate cancer cell lines corresponds to higher serum glutamate levels in the majority of prostate cancer patients compared to benign prostatic hyperplasia tissues and directly correlated with aggressiveness [21, 38, 39]. In breast cancer, human breast cancer cell line, MDA-MB-231 cells secrete glutamate corresponds to higher tissue glutamate levels [6]. In both prostate and breast cancer, excess glutamate contributes to bone metastasis [35].

To our knowledge, a connection between glutamate and VGSCs in breast cancer has never been reported. This study was designed to preliminary examine if such connection exists.

## Methods

### Cell culture

The aggressive human breast cancer cells, MDA-MB-231 which overexpresses VGSCs (nNav1.5), less aggressive human breast cancer cells, MCF-7 which lacks VGSC expression and non-cancerous human breast epithelial cell, MCF-10A were used in this study (ATCC, USA). The MDA-MB-231 and MCF-7 cells were cultured in Dulbecco’s modified Eagle’s medium (DMEM) (Nacalai, Japan) supplemented with 5% foetal bovine serum and 4 mM l-glutamine and maintained at 37 °C in a 5% CO_2_ and humidified atmosphere. The DMEM variant, DMEM-F12 was used for the MCF-10A supplemented with 5% horse serum, 20 ng/ml epidermal growth factor (EGF), 10 µg/ml insulin, 100 µg/ml hydrocortisone, and 10 ng/ml cholera toxin.

### Pharmacology

Tetrodotoxin (TTX) (Sigma-Aldrich, USA), an established VGSC-blocker was used to inhibit nNav1.5 activity and mRNA expression. After purchase, it was reconstituted in citrate buffer at 1.0 mM stock concentration. Treatment concentration of 10 µM was used on MDA-MB-231 for channel blocking effects [4, 28]. The TTX was stored in − 20 ºC until required. Dehydrated riluzole was purchased and reconstituted according to the manufacturer’s instruction (Merck, USA) which was dissolved in DMSO and dH_2_O into a stock concentration of 1.0 mM, before subsequently stored in − 20 °C until required. Treatment concentration used on MDA-MB-231 was derived from 3-[4, 5-dimethylthiazol- 2-yl]-2, 5diphenyltetrazolium bromide (MTT) assay.

### MTT assay

3 × 10^4^ cells were plated in a 96-well plate and incubated for 24 h prior to any treatment (24, 48 and 72 h). Each treatment was done in triplicate and the medium was refreshed every 24 h. The medium was removed and 100 µl of fresh DMEM and 10 µl of 12 mM of MTT were added to each well. Then, the plate was incubated at 37 °C for 4 h. After incubation period, 85 µl of MTT solution was removed and 50 µl of 100% dimethyl sulfoxide (DMSO) was added carefully to the well. The plate was incubated at 37 °C for 10 min and the absorbance was measured at 540 nm using a spectrophotometer.

#### SiRNA-mediated nNav1.5

Knockdown was conducted in order to investigate the effects of silencing nNav1.5 expression in MDA-MB-231 cells (which expressed the most significant nNav1.5 mRNA upregulation) on the concentration of glutamate. SiRNA on MDA-MB-231 cells were produced via transient transfection, whereby the siRNA sequences against nNav1.5 and controls were acquired commercially (SMARTpool siRNA reagents from Dharmacon). 3 × 10^4^ cells of MDA were seeded in a well of 24-well plate. The cells were incubated overnight. Before starting the treatment on the next day, working concentration of siRNA from a 1 µM stock resuspension was prepared/ by adding 6 µl of it into 44 µl of serum free media in a tube. A transfection solution was prepared by adding 3 µl of transfection reagent (Polyplus-transfection SA, France) into 47 µl of serum free media in another tube. Both tubes were slightly vortexed. The transfection solution was later added into the siRNA suspension and was vortexed slightly and then left at room temperature for 5 min. The cells that were incubated in the wells overnight was removed of old media 500 µl of new media added. The siRNA transfection solution was then added into the well and incubated at 37 °C for 5 h before the media was changed**.** To confirm the success of nNav1.5 knockdown, the gene in expression in the knock downed cells were measured using qPCR.

### RNA extraction and cDNA synthesis

Total RNA from the cell lines were extracted using sepasol-chloroform (Nacalai Tesque, Japan). The purity of RNA was assessed by observing the ratio of absorbance 260/280 nm and 260/230 nm in Nanodrop software. Total isolated RNA (1000 ng) was transcribed to cDNA using reverse transcription kit (Toyobo, Japan), with genomic DNA remover included by the company as component of the reverse transcription solution preparation.

#### Quantitative real-time polymerase chain reaction (qRT-PCR)

Real-time PCR was performed using SensiFAST SYBR Hi-ROX kit according to manufacturer’s protocol (Bioline, UK). Sequence primers used were as follows: β-actin forward, ATTGCCGACAGGATGCAGAAG-3′ and reverse, 5′-AGAAGCATTTGCGGTGGACG-3′ and nNav1.5 forward, 5′-CTGCACGCGTTCACTTTCCT-3′ and reverse, 5′-GACAAATTGCCTAGTTTTATATTT-3. Quantitative real-time was performed using ABI Prism 7000 Sequence Detection System (Life Technologies, USA) and the amplification conditions were as follows: initial activation for 10 min at 95 °C for one cycle, 10 s at 95 °C and 30 s at 60 °C for 34 cycles. C_t_ values of target genes were normalised to β-actin and the relative mRNA expression of target genes were calculated using the 2^−ΔΔCt^ method [30].

### Glutamate assay

Glutamate concentration in the cell supernatant (exogenous) and endogenous was measured using a fluorometric assay in a 96-well plate format. Briefly, glutamate standard and the samples were prepared according to manufacturer’s protocol (Abcam, USA). The samples were diluted to several dilutions to ensure the readings fall within the standard value range. The measurement was made using a flourescence microplate reader at  Ex/Em = 540/590 nm.

### Invasion assay

2 × 10^3^ cells were placed inside wells containing the 3D culture matrix (Cultrex, USA). The plate was centrifuged at 200×*g* for 3 min in a swing bucket rotor centrifuge (Thermo Fisher, USA) at room temperature. The plate was incubated at 37 ºC, 5% CO_2_ for 72 h. Image were taken under a compound microscope (Leica, Germany) every subsequent 24 h for 7 days. ImageJ imaging software [32] was used to analyse the size and invasion projection.  

#### Data analysis

Results are shown as the means ± SEM. Statistical evaluations were made using unpaired Student's *t* test (GraphPad Prism 9).

## Results

### Higher expression of nNav1.5 mRNA corresponds with elevated exogenous and endogenous glutamate level in aggressive human breast cancer cell line MDA-MB-231 cells

**Fig. 1:**
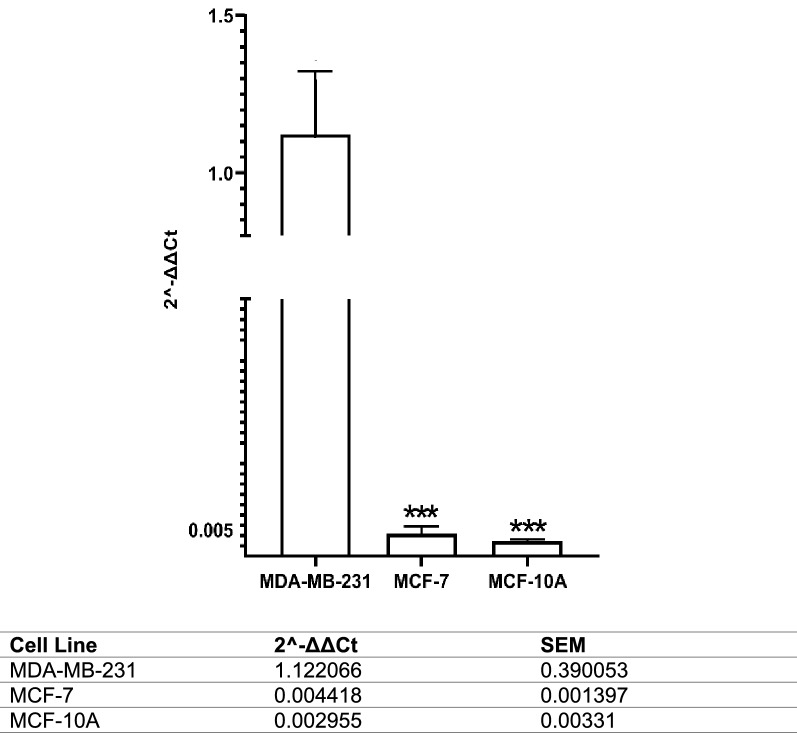
Comparison between nNav1.5 mRNA expression in human breast cancer cell lines; aggressive MDA-MB-231, less aggressive MCF-7 and the non-cancerous, MCF-10A. 2-step real-time PCR was conducted to measure the mRNA expression and 2^−ΔΔCt^ was used for the semi-quantitative analysis. Data presented as mean ± SEM n = 3 and, unpaired Student’s *t* test between MCF-7 and MCF-10A versus MDA-MB-231 *** indicate, p < 0.001

The expression of nNav1.5 mRNA in aggressive human breast cancer cell line, MDA-MB-231 was compared to less aggressive human breast cancer cell line, MCF-7 and non-cancerous human breast epithelial cell line, MCF-10A. mRNA expression level of nNav1.5 was significantly lower in MCF-7 and MCF-10A compared to MDA-MB-231. Accordingly, 2^−ΔΔCt^ value in MDA-MB-231 was 1.122 ± 0.39 (p < 0.001), while in MCF-7 and MCF-10A was 0.004 (p < 0.001) and 0.003 (p < 0.001), respectively (Fig. 1).

**Fig. 2:**
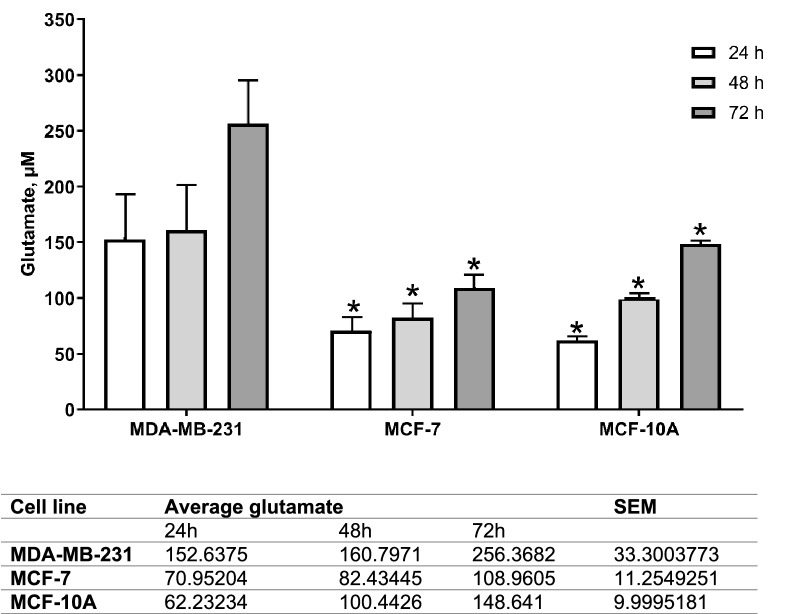
Comparison between basal exogenous glutamate concentration in human breast cancer cell lines; aggressive MDA-MB-231 and less aggressive MCF-7. The (*) on the MDA-MB-231 results show a significant difference compared to MCF-7 exogenous glutamate. Data presented as mean ± SEM n = 3 and, unpaired Student’s *t* test between MCF-7 and MCF-10A versus MDA-MB-231 * indicate, p < 0.05

Fluorometric reading for glutamate in the supernatant (exogenous glutamate) of MDA-MB-231 at 24, 48, and 72 h of culture were higher than MCF-7 and MCF-10A. When calculated using standard curve, at 24 h, exogenous glutamate level for MDA-MB-231 was 152.637 ± 33.30 µM (p < 0.05) of glutamate, and for MCF-7 and MCF-10A, 70.950 ± 11.25 (p < 0.05) and 62.232 ± 10.00 µM (p < 0.05), respectively. After 48 h, supernatant from all three cell lines showed an increase fluorometric reading and MDA-MB-231 distinctively secreted higher exogenous glutamate, 160.797 ± 33.300 µM (p < 0.05), than MCF-7 and MCF-10A, 82.434 ± 11.25 (p < 0.05), and 100.443 ± 10.00 µM (p < 0.05), respectively. Finally, at 72 h, exogenous glutamate secreted by MDA-MB-231 peaked at 256.368 ± 33.30 µM (p < 0.05), MCF-7 at 108.961 ± 11.25 (p < 0.05), and MCF-10A at 148.641 ± 10.00 µM (p < 0.05) (Fig. 2).

**Fig. 3:**
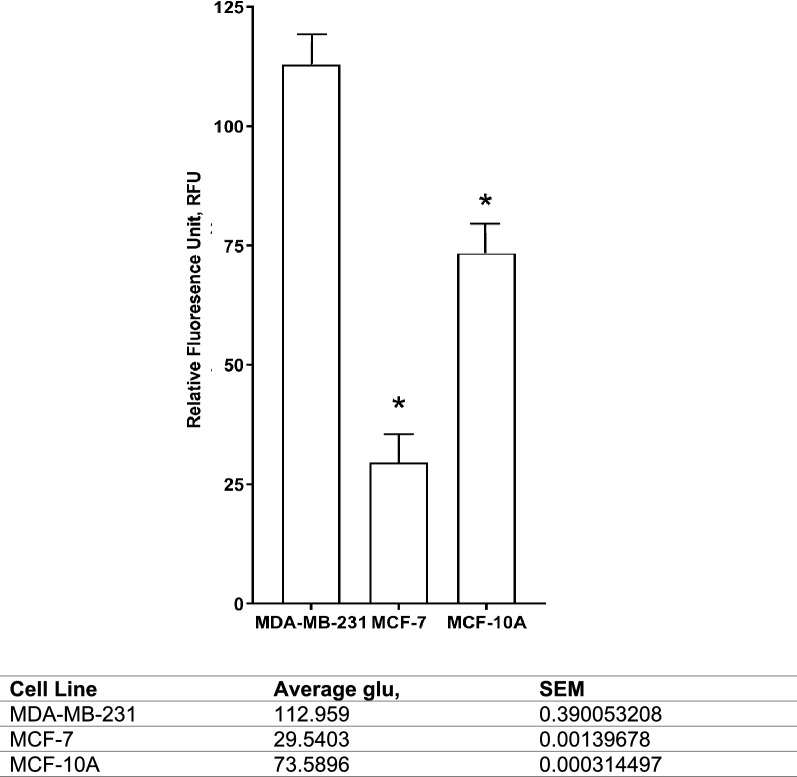
Comparison between basal endogenous glutamate concentration in human breast cancer cell lines; aggressive MDA-MB-231 and less aggressive MCF-7. The (*) on the MDA-MB-231 results show a significant difference compared to MCF-7 endogenous glutamate (p < 0.05). Data presented as mean ± SEM n = 3 and, unpaired Student’s *t* test between MCF-7 and MCF-10A versus MDA-MB-231* indicate, p < 0.05

**Fig. 4:**
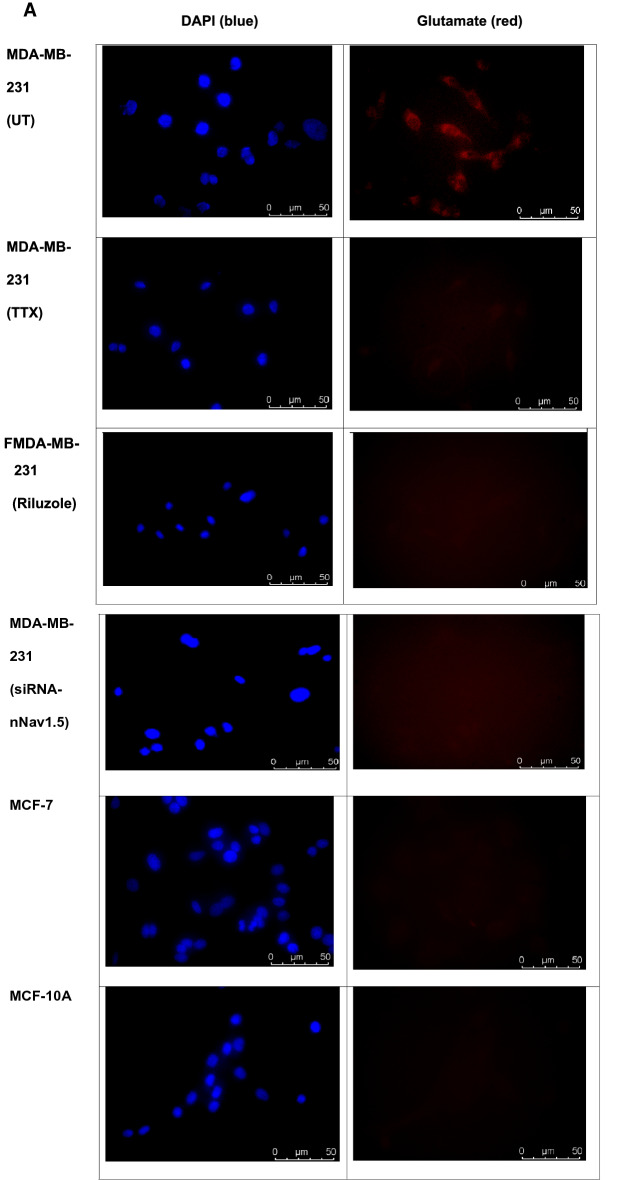

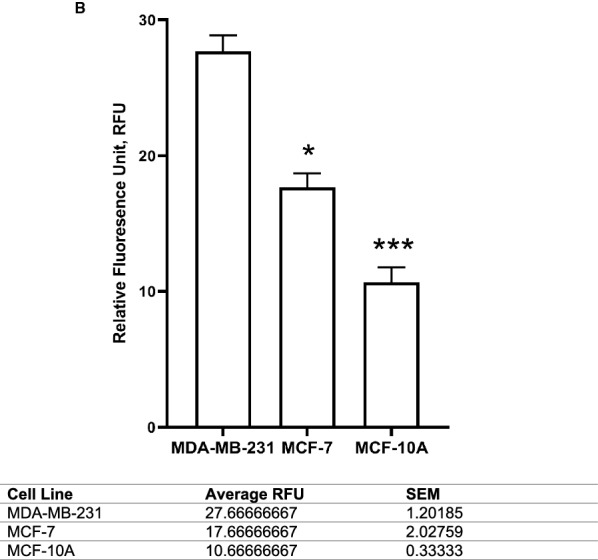
Comparison between basal glutamate concentration in human breast cancer cell lines; aggressive MDA-MB-231, less aggressive MCF-7 and the non-cancerous, MCF-10A under immunofluorescence microscopy. **A** Qualitative measurement of glutamate under fluorescence microscope. **B** Semi-quantitative measurement of glutamate relative intensity using Leica Application Suite X (LAS X) software. Data presented as mean ± SEM, n = 3 and * indicate, p < 0.05 and ***, p < 0.001

Endogenous glutamate in all cell lines was measured using fluorometric assay and fluorescence microscopy. At the end of the 72 h period from where the culture media were collected for exogenous glutamate reading, the endogenous glutamate were determined whereby again, MDA-MB-231 contained highest significant amount of endogenous glutamate at 112.959 ± 0.39 µM (p < 0.05), followed by MCF-7 at 29.540 ± 0.001 µM (p < 0.05), and MCF-10A, 73.590 ± 0.003 µM (p < 0.05) (Fig. 3). Presence of endogenous glutamate was clearly visible in a red dye under fluorescence microscopy at 400× magnification but almost not visible in MCF-7 and MCF-10A (Fig. 4A). Consequently, when the intensity of the red dye was quantified, presence of glutamate inside MDA-MB-231 was significantly highest at 27.667 ± 1.20 RFU (p < 0.05), MCF-7 at 17.667 ± 2.03 (p < 0.05), 10.667 ± 0.33 (p < 0.05) (Fig. 4B).

### Level of exogenous and endogenous glutamate in TTX-treated MDA-MB-231 cells was reduced followed by invasion suppression and downregulation of nNav1.5 expression

**Fig. 5:**
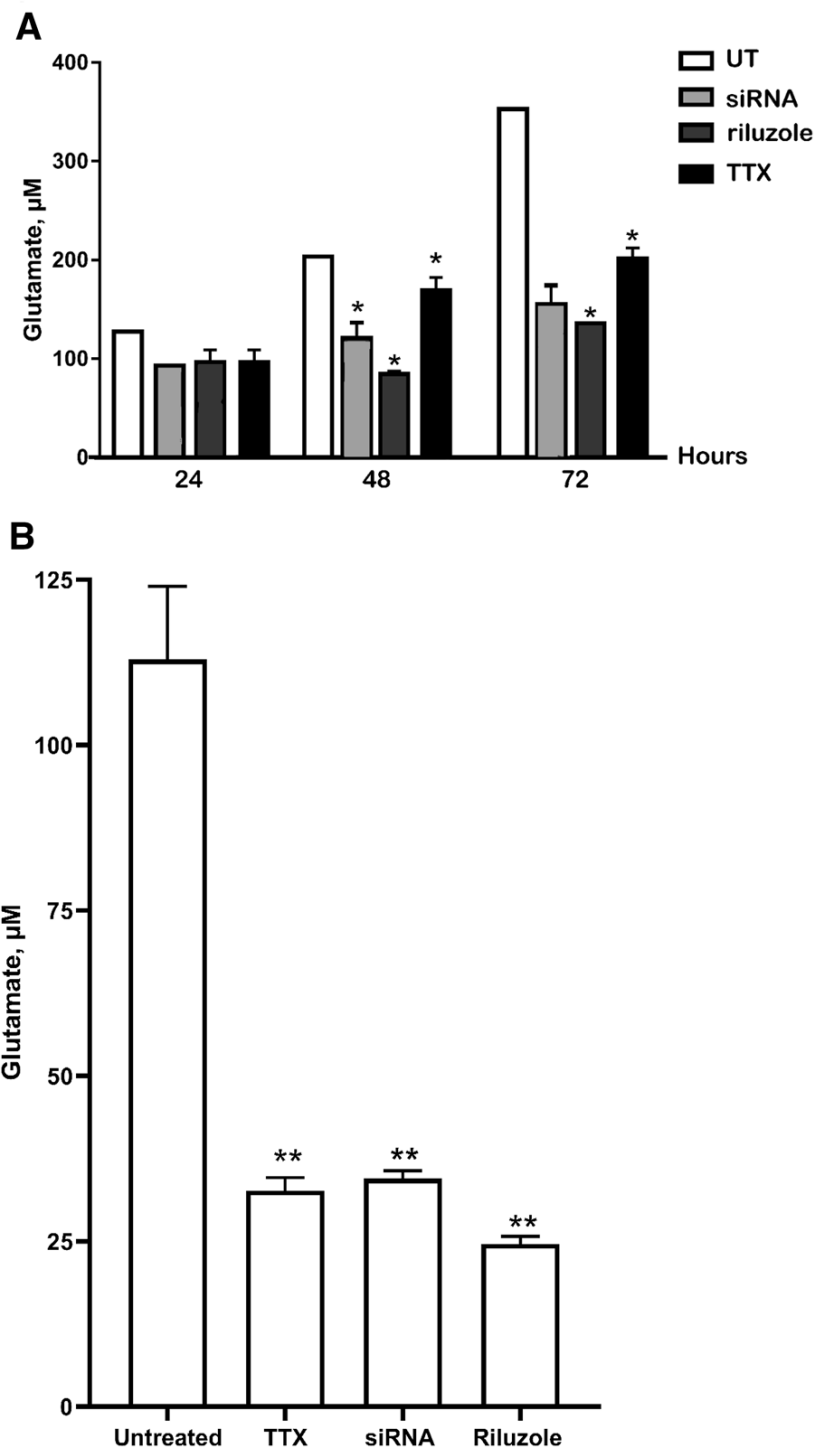
Effect of TTX, a known channel-blocking drug, on endogenous and exogenous glutamate concentration of highly metastatic MDA-MB-231 using fluorometric glutamate assay. **A** Exogenous glutamate concentration in aggressive MDA-MB-231 after treated with riluzole, TTX and siRNA-nNav1.5 compared to untreated control measured in culture supernatant after 24 h of treatment. Data presented as mean ± SEM (n = 3) and, statistical analysis used was unpaired Student’s *t* test where (*) indicate, p < 0.05. The (*) indicate significant difference of TTX, siRNA-nNav1.5 and riluzole compared to untreated control. **B** Comparison of exogenous glutamate of MDA-MB-231 between untreated and after treated with riluzole, TTX and siRNA-nNav1.5. The (*) on the treated results show a significant difference compared to untreated endogenous glutamate (p < 0.05). Data presented as mean ± SEM n = 3 and, unpaired Student’s *t* test between untreated versus treated MDA-MB-231* indicate, p < 0.05

The exogenous glutamate level in MDA-MB-231 cells was reduced significantly by 10 µM of TTX after 24 h, with untreated at 120.637 ± 33.30 µM (p < 0.05) but TTX-treated at 98.761 ± 12.33 µM (p < 0.05). After 48 h, exogenous glutamate level of untreated MDA-MB-231 was 200.797 ± 33.30 µM (p < 0.05) but TTX-treated, 172.563 ± 23.34 µM (p < 0.05). Finally, after 72 h, untreated MDA-MB-231 peaked at 356.368 ± 33.30 µM (p < 0.05) but TTX-treated at 188.341 ± 14.34 µM (p < 0.05) (Fig. 5A).

**Fig. 6:**
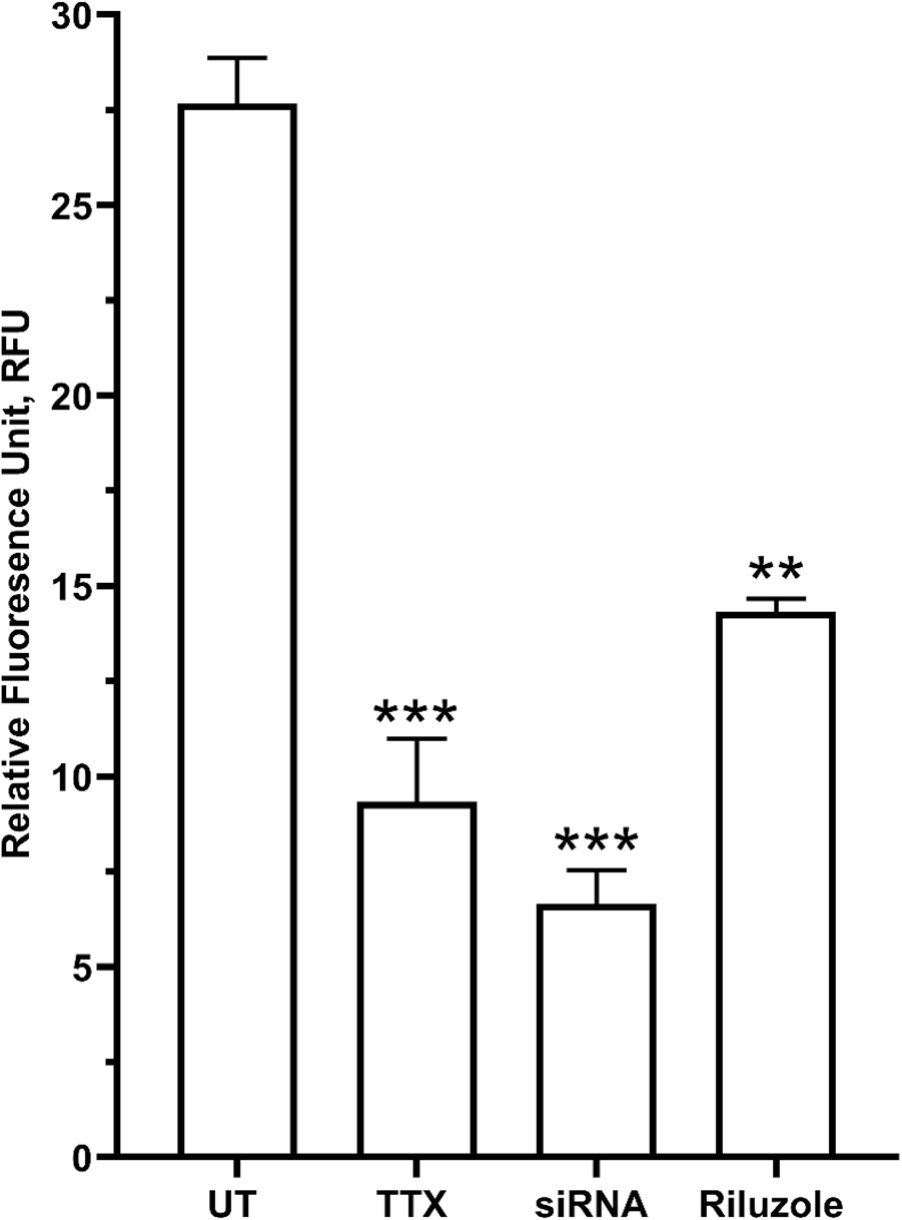
Comparison between glutamate concentration in the aggressive human breast MDA-MB-231, under untreated and TTX-treated media under immunofluorescence microscopy with semi-quantitative measurement of glutamate relative intensity using Leica Application Suite X (LAS X) software. Data presented as mean ± SEM, n = 3 and *** indicate, p < 0.001

Similar observation was obtained for endogenous glutamate with TTX-treated MDA-MB-231, significantly reduced to 43.365 ± 0.24 µM (p < 0.05) from the untreated MDA-MB-231, 112.959 ± 0.390 µM (p < 0.05) (Fig. 5B). When observed under fluorescence microscope, intensity of the red dye which indicate signal for endogenous glutamate in TTX-treated MDA-MB-231 was significantly less visible (Fig. 4A) which was reduced to 9.333 ± 1.20185 RFU (p < 0.05) at 400× magnification compared to untreated MDA-MB-231, 27.667 ± 1.2085 RFU (p < 0.05) (Fig. 6).

**Fig. 7:**
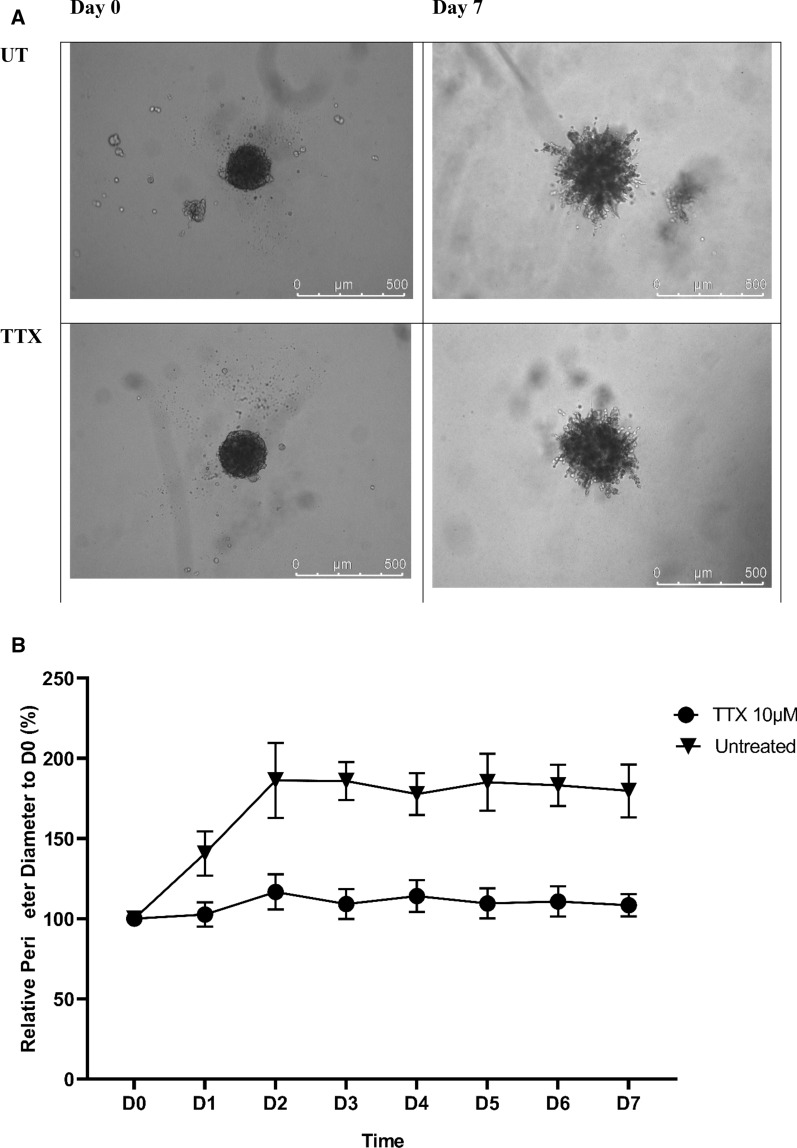
Comparison between nNav1.5 mRNA expression in the aggressive human breast MDA-MB-231, under untreated and after treated with riluzole, TTX and siRNA-nNav1.5. 2-step real-time PCR was conducted to measure the mRNA expression and 2^−ΔΔCt^ was used for the semi-quantitative analysis. Data presented as mean ± SEM n = 3 and, unpaired Student’s *t* test between untreated versus treated MDA-MB-231 *** indicate, p < 0.001

Reduction of endogenous and exogenous glutamate level by TTX diminished the ability of the cells to invade the surrounding matrix in the invasion assay (Fig. 7A, B). Briefly, high invasion spike was observed from day 1 until its peaked in size at day 3 in untreated MDA-MB-231 and later maintained its diameter and projections until the end at day 7. Whilst for TTX-treated cells diameter maintained throughout the 7 days experiments (Fig. 7B).

**Fig. 8:**
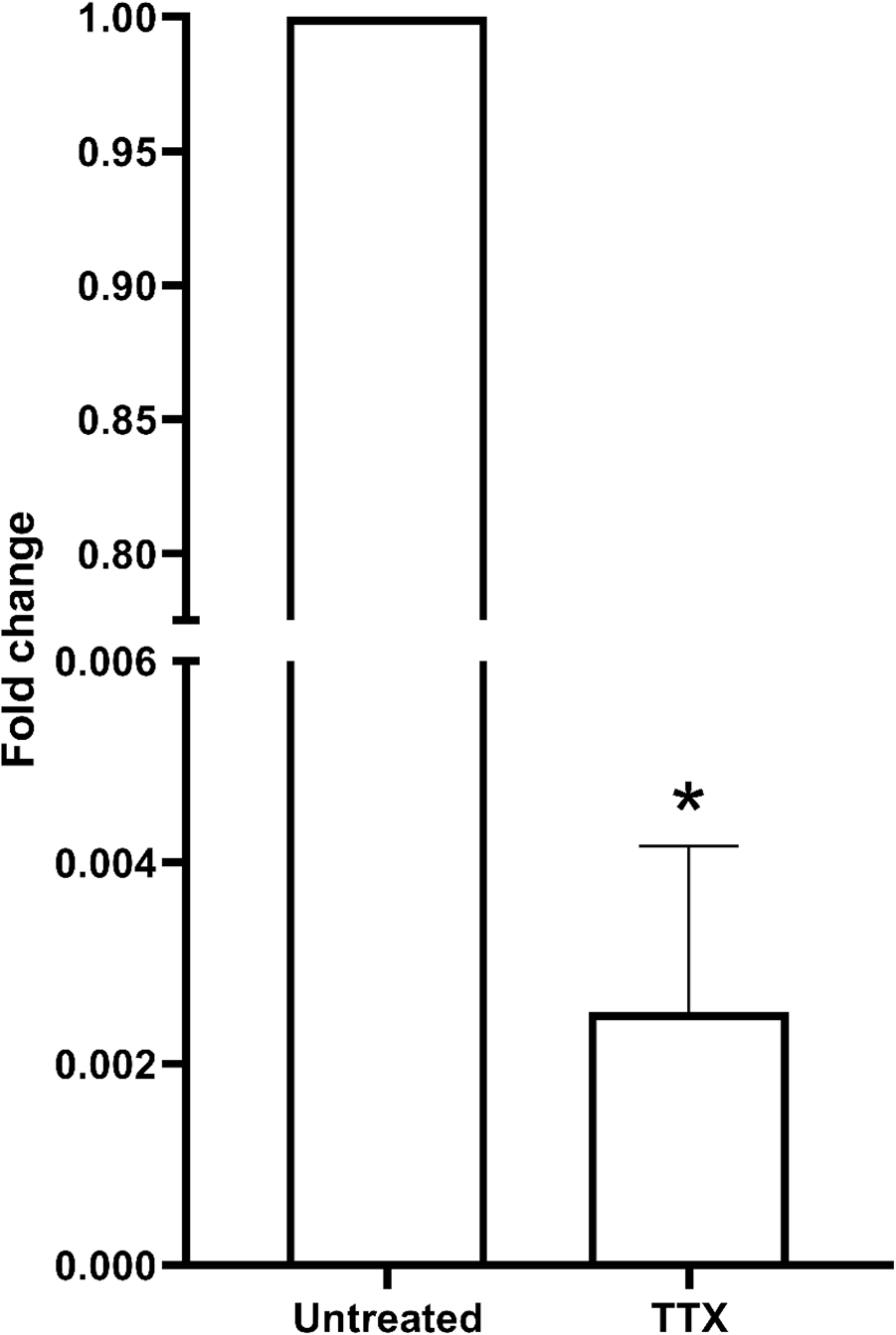
The 3D spheroid culture of MDA-MB-231 in the invasion gel matrix. **A** The image of MDA-MB-231 spheroid at day 0 and day 7 in untreated and TTX treated media. **B** The graph of the invasion rate of MDA-MB-231 untreated and under the treatment of TTX calculated by ImageJ imaging software. Data presented as mean ± SEM n = 3 and, unpaired Student’s *t* test between untreated versus treated MDA-MB-231 indicate, p < 0.05

Suppression of invasion was affirmed after analysis of nNav1.5 mRNA revealed significant downregulation in TTX-treated cells. Briefly, 2^−ΔΔCt^ for untreated MDA-MB-231 cells was 1.122 ± 0.390 whilst for TTX-treated cells, 4.598 × 10^–3^ ± 0.002 (Fig. 8).

## Discussion

Multiple lines of evidence have indicated clinical significance of elevated serum, plasma and tissue glutamate in the prognosis of prostate, pancreatic, lung and breast cancer [6, 19, 21, 40]. In separate studies, VGSC expression in respective cell lines of these glutamate-enrich tumours is known [14, 18, 29]. nNav1.5 is a potent metastatic marker for breast cancer [1, 10]. Elevated levels of endogenous and exogenous glutamate in aggressive human breast cancer cells, MDA-MB-231 which expresses nNav1.5 compared to the less aggressive human breast cancer cells, MCF-7 which lacks nNav1.5 conforms with the hypothesis that high glutamate and expression of nNav1.5 is interconnected in breast cancer. Unexpectedly, MCF-10A had higher glutamate than MCF-7 even if it is significantly lower than the subject of interest, MDA-MB-231. MCF 10A, as reported by the American Type Culture Collection (ATCC), is a non-cancerous cell. But it shows certain tumour markers such as basal-like phenotype [31]. This is due to the process of immortalisation of the cell line from primary culture itself. Because of this, no secondary cell line are to be considered normal post-immortalisation such as telomere lengthening process whereby the immortal cells tend to derive chromosome abnormality [41] resulting in results obtained. Other research also points out the relevance of MCF-10A as non-tumorous breast epithelial cell-line model and it was due to increased karyotyping in the cell lines causing certain proteins to be expressed higher [27, 31].

In examining the mode of connection between these two, MDA-MB-231 cells was treated with TTX (specific blocker for VGSC), a common ‘tool’ used to study VGSC in excitable cells and in the study of ‘proof-of-concept’ for the role of VGSC in cancers. Earlier, the use of TTX has helped researchers to understand the critical method role of VGSC in regulating excitotoxic glutamate release in several ischemic induced in vitro and in vivo models where TTX exerted neuroprotective effects against glutamate‑induced cell toxicity via VGSC blockade [23]. Concordantly, endogenous, and exogenous glutamate level was significantly reduced in TTX-treated MDA-MB-231 cells, revealing for the first time the role of nNav1.5 expression in regulating glutamate level in aggressive breast cancer. Treatment with TTX also led to significant downregulation of nNav1.5 gene expression followed by inhibition of the cell invasion. The observed cell invasion suppression in this study is unrelated to any effects on cell viability as the TTX concentration used (10 µM) was sub-lethal—20× lower than the IC_50_, > 200 µM. These observations are in accordance to study that showed TTX blockade of the channel’s pore, prevented the amount of Na^+^ entering the cells thus interfered with the positive autoregulation of the channels expression at the plasma membrane which led to suppression of metastatic ability of MDA-MB-231 [8].

At the meantime, glutamate is a known potent necrosis factor during neuro-excitotoxicity [2, 33] and in glioblastoma [25]. Subsequently, tumour aggressiveness is exploiting excitotoxicity mechanisms to kill (necrotising) surrounding cells to create way for metastasis [37]. In the case of breast cancer, metastasis to bone is a feature of aggressive breast cancer cells that secrete high levels of glutamate [12, 13]. The 3D spheroid invasion assay was used in this study to replace wound healing and Matrigel assays to provide more accurate insights on the invasion since spheroid have shown to have higher cell proliferation, migration, and invasion rate [3]. Regardless, the use of TTX have been shown to reduce the effects of wound healing and Matrigel assays according to previous studies [14, 15]. Besides being used as a preferred in vitro model for nNav1.5 expression and glutamate secretion, MDA-MB-231 cells is also an established cell line used for development of a breast cancer bone metastases model, primarily to long bones, spine, jaw and lungs [42]. nNav1.5, expression and activity in these cells has been demonstrated to regulate the pH-dependent activity of cathepsins B and S, a type of proteinases that degrade extracellular matrix [5, 11, 16]. Interestingly, emerging lines of evidence are also pointing to the pro-metastatic role of cathepsin B in bone metastasis of breast cancer [26].

With regards to VGSCs in breast cancer, a wealth of data has now confidently demonstrated that Nav1.5 and its splice variant nNav1.5 offers potential value as metastatic tumour markers in the diagnosis (including prognosis), and therapies of the disease. Previously, the significance of therapeutic values for nNav1.5 and glutamate has been reported separately. nNav1.5 gene and protein expression in breast cancer tissues has been reported elsewhere [43] and now its value as prognostic and predictive marker is currently being evaluated. In doing so, works on development and characterisation of antibodies against nNav1.5 has been reported. In this regard, a polyclonal rabbit antibody, NESOpAb was generated to specifically recognise the neonatal splice form of Nav1.5 and excluding the adult counterpart [7] that utilise on their discovery of the 7 divergence of amino acid in the former since the divergence is significant enough to have its own specific antibody. Further achievement from our inhouse project managed to develop a novel monoclonal mouse antibody for nNav1.5, 4H8 [36]. This benefits directly for future therapeutic development with better specificity on a single epitope, allowing mass production of diagnostic tools for nNav1.5. As for glutamate, including the fact that nNav1.5 is correlated with metastatic breast cancer and in other cases also shows significance of blocking nNav1.5 on another molecule such as Major Histocompatibility Complex (MHC) Class I [24].

## Conclusions

In summary, our study represents a unique connection between nNav1.5 expression and glutamate in aggressive breast cancer cells. Importantly, our findings raise to new understandings and opportunity to a new therapeutic strategy to combat metastasis which is the major cause of mortality for breast cancer patients.

## Data Availability

All relevant data are within the paper.

## Abbreviations

%: Percentage
× g: Times gravity
®: Registered
°C: Degree Celsius
Ab: Antibody
ATCC: American Type Cell Culture
Ca2+: Calcium ion
CO2: Carbon dioxide
Ct: Cycle threshold
dH_2_O: Distilled water
DMEM: Dulbecco’s modified Eagle’s medium
EGF: Epidermal growth factor
H+: Hydrogen ion
mAb: Monoclonal antibody
MHC: Major Histocompatibility Complex
ml: Millilitre
mM: Mili molar
mRNA: Messenger ribonucleic acid
Na+: Sodium ion
Nav: Voltage-gated sodium channel
NESOpAb: Nav1.5 polyclonal antibody
ng: Nano gram
nm: Nano metre
nNav1.5: Neonatal Nav1.5
pAb: Polyclonal antibody
pH: Potential of hydrogen
qPCR: Real-time polymerase chain reaction
qRT-PCR: Quantitative real-time reverse transcription polymerase chain reaction
RFU: Relative fluorescence unit
SEM: Standard error of mean
TTX: Tetrodotoxin
VGSC: Voltage-gated sodium channel
α: Alpha
β: Beta
β-actin: Beta actin
μ: Micro
μg: Microgram
μl: Microlitre
μM: Micro molar

## References

[1] Angus M, Ruben P (2019). Voltage gated sodium channels in cancer and their potential mechanisms of action. Channels.

[2] Belov Kirdajova D, Kriska J, Tureckova J, Anderova M (2020). Ischemia-triggered glutamate excitotoxicity from the perspective of glial cells. Front Cell Neurosci.

[3] Boo L, Ho WY, Ali NM, Yeap SK, Ky H, Chan KG, Yin WF, Satharasinghe DA, Liew WC, Tan SW, Ong HK, Cheong SK (2016). MiRNA Transcriptome Profiling of Spheroid-Enriched Cells with Cancer Stem Cell Properties in Human Breast MCF-7 Cell Line. Int J Biol Sci.

[4] Brackenbury WJ, Chioni A-M, Diss JKJ, Djamgoz MBA (2007). The neonatal splice variant of Nav1.5 potentiates in vitro invasive behaviour of MDA-MB-231 human breast cancer cells. Breast Cancer Res Treat.

[5] Brisson L, Gillet L, Calaghan S, Besson P, Guennec JY, Roger S, Gore J (2011). Na v 1.5 enhances breast cancer cell invasiveness by increasing NHE1-dependent H efflux in caveolae. Oncogene.

[6] Budczies J, Pfitzner BM, Györffy B, Winzer KJ, Radke C, Dietel M, Fiehn O, Denkert C (2015). Glutamate enrichment as new diagnostic opportunity in breast cancer. Int J Cancer.

[7] Chioni A-M, Fraser SP, Pani F, Foran P, Wilkin GP, Diss JKJ, Djamgoz MBA (2005). A novel polyclonal antibody specific for the Nav1.5 voltage-gated Na+ channel ‘neonatal’ splice form. J Neurosci Methods.

[8] Chioni AM, Shao D, Grose R, Djamgoz MBA (2010). Protein kinase A and regulation of neonatal Nav1.5 expression in human breast cancer cells: Activity-dependent positive feedback and cellular migration. Int J Biochem Cell Biol.

[9] Diaz D, Delgadillo DM, Hernández-Gallegos E, Ramírez-Domínguez ME, Hinojosa LM, Ortiz CS, Berumen J, Camacho J, Gomora JC (2007). Functional expression of voltage-gated sodium channels in primary cultures of human cervical cancer. J Cell Physiol.

[10] Djamgoz MBA, Fraser SP, Brackenbury WJ (2019). *In vivo* evidence for voltage-gated sodium channel expression in carcinomas and potentiation of metastasis. Cancers.

[11] Dutta S, Lopez Charcas O, Tanner S, Gradek F, Driffort V, Roger S, Selander K, Velu SE, Brouillette W (2018). Discovery and evaluation of nNav15 sodium channel blockers with potent cell invasion inhibitory activity in breast cancer cells. Bioorganic Med Chem.

[12] Fazzari J, Lin H, Murphy C, Ungard R, Singh G (2015). Inhibitors of glutamate release from breast cancer cells.

[13] Fazzari J, Linher-Melville K, Singh G (2016). Tumour-derived glutamate: linking aberrant cancer cell metabolism to peripheral sensory pain pathways. Curr Neuropharmacol.

[14] Fraser SP, Diss JKJ, Chioni AM, Mycielska ME, Pan H, Yamaci RF, Pani F, Siwy Z, Krasowska M, Grzywna Z, Brackenbury WJ, Theodorou D, Koyutürk M, Kaya H, Battaloglu E, De Bella MT, Slade MJ, Tolhurst R, Palmieri C, Djamgoz MBA (2005). Voltage-gated sodium channel expression and potentiation of human breast cancer metastasis. Clin Cancer Res.

[15] Gao R, Shen Y, Cai J, Lei M, Wang Z (2010). Expression of voltage-gated sodium channel subunit in human ovarian cancer. Oncol Rep.

[16] Gillet L, Roger S, Besson P, Lecaille F, Gore J, Bougnoux P, Lalmanach G, Le Guennec JY (2009). Voltage-gated sodium channel activity promotes cysteine cathepsin-dependent invasiveness and colony growth of human cancer cells. J Biol Chem.

[17] Greco MR, Antelmi E, Busco G, Guerra L, Rubino R, Casavola V, Reshkin SJ, Cardone RA (2014). Protease activity at invadopodial focal digestive areas is dependent on NHE1-driven acidic pHe. Oncol Rep.

[18] Grimes JA, Fraser SP, Stephens GJ, Downing JEG, Laniado ME, Foster CS, Abel PD, Djamgoz MBA (1995). Differential expression of voltage-activated Na+ currents in two prostatic tumour cell lines: contribution to invasiveness *in vitro*. FEBS Lett.

[19] Herner A, Sauliunaite D, Michalski CW, Erkan M, Oliveira TD, Abiatari I, Kong B, Esposito I, Friess H, Kleeff J (2011). Glutamate increases pancreatic cancer cell invasion and migration via AMPA receptor activation and Kras-MAPK signaling. Int J Cancer.

[20] House CD, Vaske CJ, Schwartz AM, Obias V, Frank B, Luu T, Sarvazyan N, Irby R, Strausberg RL, Hales TG, Stuart JM, Lee NH (2010). Voltage-gated Na+ channel SCN5A is a key regulator of a gene transcriptional network that controls colon cancer invasion. Can Res.

[21] Koochekpour S, Majumdar S, Azabdaftari G, Attwood K, Scioneaux R, Subramani D, Manhardt C, Lorusso GD, Willard SS, Thompson H, Shourideh M, Rezaei K, Sartor O, Mohler JL, Vessella RL (2012). Serum glutamate levels correlate with gleason score and glutamate blockade decreases proliferation, migration, and invasion and induces apoptosis in prostate cancer cells. Clin Cancer Res.

[22] Li CT, Yang KC, Lin WC (2019). Glutamatergic dysfunction and glutamatergic compounds for major psychiatric disorders: Evidence from clinical neuroimaging studies. Front Psychiatry.

[23] Lysko PG, Webb CL, Yue TL, Gu JL, Feuerstein G (1994). Neuroprotective effects of tetrodotoxin as a na+ channel modulator and glutamate release inhibitor in cultured rat cerebellar neurons and in gerbil global brain ischemia. Stroke.

[24] Murtadha AH (2021). Influence of nNav15 on MHC class I expression in breast cancer. J Biosci.

[25] Noch E, Khalili K (2009). Molecular mechanisms of necrosis in glioblastoma: The role of glutamate excitotoxicity. Cancer Biol Ther.

[26] Nouh MA, Mohamed MM, El-Shinawi M, Shaalan MA, Cavallo-Medved D, Khaled HM, Sloane BF (2011). Cathepsin b: A potential prognostic marker for inflammatory breast cancer. J Transl Med.

[27] Odetallah MM (2003). Cytogenetic analysis of primary breast tumors and MCF10A cells to determine early steps of breast carcinoma. Cells.

[28] Onkal R, Djamgoz MB (2009). Molecular pharmacology of voltage-gated sodium channel expression in metastatic disease: clinical potential of neonatal Nav1.5 in breast cancer. Eur J Pharmacol.

[29] Pancrazio JJ, Viglione MP, Tabbara IA, Kim YI (1989). Voltage-dependent Ion channels in small-cell lung cancer cells. Cancer Res.

[30] Pfaffl MW, Horgan GW, Dempfle L (2002). Relative expression software tool (REST) for group-wise comparison and statistical analysis of relative expression results in real-time PCR. Nucleic Acids Res.

[31] Qu Y (2015). Evaluation of MCF10A as a Reliable Model for Normal Human Mammary Epithelial Cells. PLoS ONE.

[32] Rasband WS. ImageJ, U.S. National Institutes of Health, Bethesda, Maryland, USA. 2014. http://Imagej.Nih.Gov/Ij/.

[33] Rothstein JD, Jin L, Dykes-Hoberg M, Kuncl RW (1993). Chronic inhibition of glutamate uptake produces a model of slow neurotoxicity. Proc Natl Acad Sci USA.

[34] Seidlitz EP, Sharma MK, Saikali Z, Ghert M, Singh G (2009). Cancer cell lines release glutamate into the extracellular environment. Clin Exp Metas.

[35] Sharma MK, Seidlitz EP, Singh G (2010). Cancer cells release glutamate via the cystine/glutamate antiporter. Biochem Biophys Res Commun.

[36] Sharudin NA (2020). 65P The new mouse anti-nNav1.5 monoclonal antibody. Ann Oncol.

[37] Sontheimer H (2008). A role for glutamate in growth and invasion of primary brain tumors. J Neurochem.

[38] Sreekumar A, Poisson LM, Rajendiran TM, Khan AP, Cao Q, Yu J, Laxman B, Mehra R, Lonigro RJ, Li Y, Nyati MK, Ahsan A, Kalyana-Sundaram S, Han B, Cao X, Byun J, Omenn GS, Ghosh D, Pennathur S, Chinnaiyan AM (2009). Metabolomic profiles delineate potential role for sarcosine in prostate cancer progression. Nature.

[39] Taylora BS, Pale M, Yu J, Laxman B, Kalyana-Sundaram S, Zhao R, Menon A, Wei JT, Nesvizhskii AI, Ghosh D, Omenn GS, Lubman DM, Chinnaiyan AM, Sreekumar A (2008). Humoral response profiling reveals pathways to prostate cancer progression. Mol Cell Proteomics.

[40] Vanhove K, Giesen P, Owokotomo OE, Mesotten L, Louis E, Shkedy Z, Thomeer M, Adriaensens P (2018). The plasma glutamate concentration as a complementary tool to differentiate benign PET-positive lung lesions from lung cancer. BMC Cancer.

[41] Wenger SL (2004). Comparison of established cell lines at different passages by karyotype and comparative genomic hybridization. Biosci Rep.

[42] Wright LE, Ottewell PD, Rucci N, Peyruchaud O, Pagnotti GM, Chiechi A, Buijs JT, Sterling JA (2016). Murine models of breast cancer bone metastasis. BoneKEy Reports.

[43] Yamaci RF, Fraser SP, Battaloglu E, Kaya H, Erguler K, Foster CS, Djamgoz MBA (2017). Neonatal Nav1.5 protein expression in normal adult human tissues and breast cancer. Pathol - Res Pract.

[44] Yang M, Kozminski DJ, Wold LA, Modak R, Calhoun JD, Isom LL, Brackenbury WJ (2012). Therapeutic potential for phenytoin: Targeting Nav1.5 sodium channels to reduce migration and invasion in metastatic breast cancer. Breast Cancer Res Treatment.

